# Substitutes and Colloidal System for Vitreous Replacement and Drug Delivery: Recent Progress and Future Prospective

**DOI:** 10.3390/polym13010121

**Published:** 2020-12-30

**Authors:** Minal Thacker, Ching-Li Tseng, Feng-Huei Lin

**Affiliations:** 1 Graduate Institute of Biomedical Engineering, National Taiwan University, Daan District, Taipei 10051, Taiwan; minal.thacker11@gmail.com; 2 Graduate Institute of Biomedical Materials and Tissue Engineering, Taipei Medical University, Taipei 11031, Taiwan; 3 Institute of Biomedical Engineering and Nanomedicine, National Health Research Institutes, Miaoli County 35053, Taiwan

**Keywords:** vitreous substitute, biopolymer

## Abstract

Vitreoretinal surgeries for ocular diseases such as complicated retinal detachment, diabetic retinopathy, macular holes and ocular trauma has led to the development of various tamponades over the years in search for an ideal vitreous substitute. Current clinically used tamponade agents such as air, perfluorocarbons, silicone oil and expansile gases serve only as a short-term solution and harbors various disadvantages. However, an ideal long-term substitute is yet to be discovered and recent research emphasizes on the potential of polymeric hydrogels as an ideal vitreous substitute. This review highlights the recent progress in the field of vitreous substitution. Suitability and adverse effects of various tamponade agents in present day clinical use and biomaterials in the experimental phase have been outlined and discussed. In addition, we introduced the anatomy and functions of the native vitreous body and the pathological conditions which require vitreous replacement.

## 1. Introduction

Treatment of vitreoretinal disorders has advanced significantly in the recent years. Various materials have been proposed and evaluated over the years in order to find an appropriate vitreous substitute. Despite many years of research, a suitable long-term vitreous substitute still remains a challenge due to various drawbacks of the clinically used vitreous agents today. The ideal vitreous substitute should mimic the native vitreous in terms of both structure and function such as transparency, biocompatibility, elasticity, easy injectability, except for its liquefication and biodegradability with age [1,2]. Current vitreous substitutes are used in order to maintain certain criterias such as optical and biomechanical properties and intraocular pressure. Therefore, extensive research with biomaterials is underway taking into consideration its composition, structure and physiological properties as well to overcome the downside of the currently used tamponades.

In this review, we provide an overview of the anatomy and functions of the vitreous body, as well as the conditions requiring vitreous substitution. It majorly focuses on the newest developments in biomaterials which are either polymeric hydrogels or smart injectable hydrogels that have been experimented and researched for vitreous substitution. Finally, a future perspective of the application of colloidal and nanocarriers system in the field of vitreous has been discussed in brief.

## 2. Vitreous Body

### 2.1. Anatomy and Chemistry

**Figure 1:**
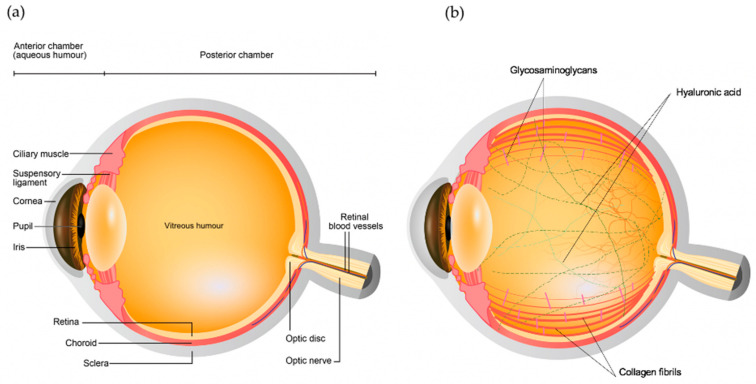
Schematic diagram of the (**a**) ocular structures and (**b**) vitreous components.

The eye primarily consists of two parts, an anterior and a posterior segment. The anterior segment is the smaller part of the eye which includes the cornea, conjunctiva, iris, pupil, aqueous humor and lens, while the posterior segment consists of sclera, choroid and retina surrounding the vitreous chamber filled with vitreous humor [3], as shown in Figure 1a. Vitreous humor is a transparent gel like matrix occupying two-third of the volume of the eye. The vitreous is highly hydrated comprising of over 98% of water, with 15–20 wt% of total water bound to proteins and Glycosaminoglycans (GAGs) [4,5]. The most abundant insoluble protein found in the vitreous is collagen, which builds a three-dimensional framework within the vitreous gel. Amongst the various collagen types present in the vitreous, type II collagen is found to be the most extensive in the vitreous (65%), followed by type IX (25%), type V/XI (10%), and type IV (<10%) [6], as shown in Table 1 and Figure 1b. On the other hand, GAGs constitute the other important component of the vitreous (Figure 1b). Three types of GAGs are present, mainly hyaluronic acid (HA) and with much lower concentration chondroitin sulfate (CS) and heparan sulfate (HS) [7]. HA is considered as a crucial element in determining the viscosity of the vitreous due to its massive size (molecular weight of 100,000–10,000,000 DA), while CS is a sulfated GAG and is generally linked to proteins as a part of a proteoglycan. It is present in the vitreous in the form of two proteoglycans: type IX collagen and versican. It helps in sustaining the structural integrity of the tissue [6,8]. Lastly, HS is present in small quantities in the vitreous and helps in maintaining adequate distance between the collagen fibrils [9]. The viscoelastic property of the vitreous is determined by the organization of hyaluronic acid between the interstices of long collagen fibrils suspended in the vitreous [10].

### 2.2. Functions of the Vitreous

#### 2.2.1. Volume, Growth and Elasticity

Vitreous is a mixture of many components including long collagen fibrils, HA, CS, HS and various ions which makes it dense and viscous. The vitreous plays a crucial role in the growth of the eye. According to the previous studies, the growth of the vitreous stimulates the growth of the eye and the retinal pigmented epithelium (RPE) through HA production which plays a vital role in this growth process [13]. The major function of the vitreous is to protect the structures of the eye and most importantly the retina from low frequency mechanical stress, vibration and friction that occurs due to the constant eye movement in everyday life. Moreover, the viscoelastic property of vitreous due to high HA content makes it an outstanding shock-absorber against physical force [14]. However, this property of vitreous does not last long due to its liquefication with age [11]. Vitreous also plays a role in the growth and development of the eyeball and also can inhibit the formation of new blood vessels. An increase in angiogenic factor such as vascular endothelial growth factor (VEGF) causes wet age-related macular degeneration (wet AMD) which leads to irreversible vision loss in elderly people. However, a group of researchers demonstrated that the release of anti-VEGF from thermo-responsive mPEG-PLGA-BOX hydrogel suppressed angiogenesis both in vitro and in vivo [15].

#### 2.2.2. Transparency and Accommodation

Transparency is the key feature of the vitreous that allows the transmission of light from ultraviolet to infrared region. Vitreous transmits ~90% of visible light towards the retina, similar to that of the aqueous [16]. Large HA molecules get attached to the collagen fibers creating a relatively large distance between the fibrils as a result of which little light scattering occurs in the vitreous. Vitreous also aids in bearing the lens capsule, thereby facilitating the accommodating process [6].

#### 2.2.3. Biological and Barrier Function

It was found out that the vitreous can act as a metabolic depot for the surrounding eye tissues. The gel like vitreous reduces the exposure of the lens to oxygen, thereby, protecting the lens from oxidative damage and cataract formation [17]. Another major role of vitreous is to act as a barrier against various biochemical molecules and cells, thereby preventing bacterial infection and the accompanied inflammation, however, few viral agents might be able to flourish causing viral infections. It also helps in inhibiting neovascularization and inflammation by being an important part of blood-ocular barrier in normal healthy eye [18,19].

## 3. Pathological Conditions Requiring Vitreous Substitution

### 3.1. Ageing

According to the studies, vitreous in Rhesus monkeys and humans tends to gradually liquefy and shrinks as age progresses under a phenomenon known as syneresis as compared to the other mammals such as cats, dogs, rabbits, cattle whose vitreous undergoes relatively small changes with the increasing age [20,21]. In view of the existing literature, the liquefication of vitreous is mainly due to the degradation of type IX collagen. Type IX collagen typically aids in preventing the collagen type II fibrils to adhere with each other by forming a layer of coating on the surface of type II collagen fibrils. With the increasing age, more and more type IX collagen get degraded and hence the coating on the collagen fibrils diminishes, as a result of which the vitreous collagen fibrils tend to form aggregates. These aggregated clusters later result in the expulsion HA that were previously located in the interstices of the collagen fibrils. The dissociation of hyaluronan from collagen fibrils results in the formation of a liquid phase rendering in the liquefication of the vitreous [22]. According to the reports, the liquefication of the vitreous in people over the age of 80 is about 50% [23,24].

### 3.2. Posterior Vitreous Detachment

**Figure 2:**
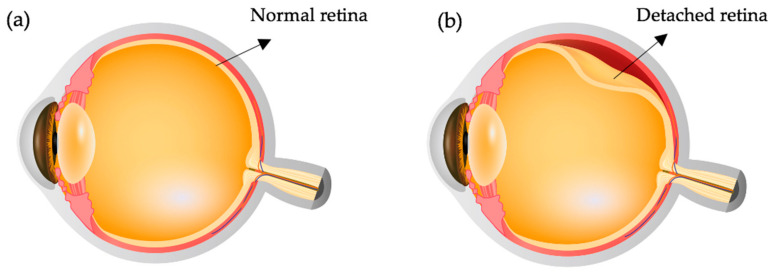
(**a**) Healthy eye (**b**) Retinal detachment.

The liquification of the vitreous in the due course may lead to a degenerative process known as posterior vitreous detachment (PVD). It is characterized by the detachment of the vitreous cortex from the retina [25]. PVD can eventually lead to retinal detachment (RD) (Figure 2) by constant pulling and tugging by the vitreous in the retinal regions where the vitreous is tightly attached [26]. The most common symptoms of PVD includes floaters and photopsias (lightning streaks) due to difference in scattering of light in the vitreous through its liquefied and gel portions [27]. The condensed free-floating collagen fibrils in the liquefied vitreous may also induce glares [11]. Hence, RD and PVD would require an internal vitreous tamponading agent.

### 3.3. Diabetic Retinopathy

Diabetes is rising worldwide with an alarming rate, threating public health. Diabetic patients can have an eye disease called diabetic retinopathy (DR). Millions of patients are affected with diabetic retinopathy (DR) with varying severity. DR is characterized by the damage of blood vessels in the retina of diabetic patients due to the elevated glucose levels in their vitreous. These blood vessels tend to swell up and leak or can even get closed, stopping the blood flow [28]. Therefore, cases like these might require artificial substitutes like silicone oil or gas [29].

### 3.4. Ocular Trauma

Certain injury causes ocular trauma which results in substantial damage to the eyes including damage to the vitreous. Such injuries require vitreo-retinal surgeries in order to repair the eye structure using internal tamponading agents [30].

## 4. Currently Used Vitreous Substitutes in Clinics

The most commonly used tamponading agents to be used as suitable vitreous substitutes over the years ranges from gases to liquids particularly expansile gases and silicone oil. Even though they impose great advantages such as chemical inertness and good optical clarity, these agents are far from being the ideal substitutes accompanying many limitations and drawbacks [29]. Therefore, the following section will summarize the characteristics and limitations of the currently available vitreous substitutes classified on the basis of their molecular state into gas-based or air and liquid-based substitute (Table 2).

### 4.1. Gas-Based Substitutes

#### 4.1.1. Air

Air was the first gas to be used as a tamponading agent to reattach the retina in 1911 by Ohm [31]. It is chemically inert, inexpensive, colorless, non-toxic and most importantly abundant in nature and requires no secondary surgery for its removal as its easily diffusible into the blood circulation. Due to its easily absorbable nature, the residence time of air in the chamber is approximately few days which is a major limitation as the tamponading effect would not last long [32,33]. Additionally, another major drawback is its refractive index (1.0008) which is significantly less as compared to the natural human vitreous (~1.33) which results in complete reflection of light and poor vision [34]. Therefore, its use as a vitreous substitute is limited, however it is used at the end of vitrectomy procedure in pneumatic retinopexy and also at the time of unavailability of other substitutes [35].

#### 4.1.2. Other Gases

The widespread use of expansile gases began in early 1970s. Norton in 1973 first used Sulphur hexafluoride (SF_6_) as a vitreous substitute [36] and found it to be better than air in terms of heaviness and relatively longer intra-vitreal residence time of around 18 days. Currently, the most commonly used gases in the vitreoretinal disease treatment are sulfur hexafluoride (SF_6_) and perfluoropropane (C_3_F_8_) [37]. Both these gases are widely used due to its colorless, odorless and nontoxic characteristics. Moreover, due to their high surface tension and diffusion of other gases into these gases from the circulation they are able to sustain their tamponade effect in the vitreous chamber [32,38]. SF_6_ expands twice its volume within 1 to 2 days after injection and exert effect in the vitreous cavity for 1–2 weeks, while C_3_F_8_ after injection expands to four times its volume within 3–4 days and lasts in the vitreous cavity for 6–8 weeks. In 1993, these gases were approved by the U.S. Food and Drug Administration for their use in pneumatic retinopexy due to their longer residence time as compared to the air [11,39].

The tamponade effect of these gases depends on the position of the gas bubbles inside the cavity which is limited to the upper portion of the gas bubble, therefore, awkward face down position is required for several days after administration in order to exert the tamponading effect on the inferior retina [40,41]. It is also recommended to avoid air travels and high-altitude places post-surgery for several weeks due to the expansible nature of these gases [42]. Additionally, severe side effects include cataract formation, increased intraocular pressure during the surgery or few days post administration which might result in damage to the optic nerve [43]. Therefore, these agents are not an ideal long-term substitute of vitreous.

### 4.2. Liquid-Based Substitutes

#### 4.2.1. Physiological Solutions

**Figure 3:**
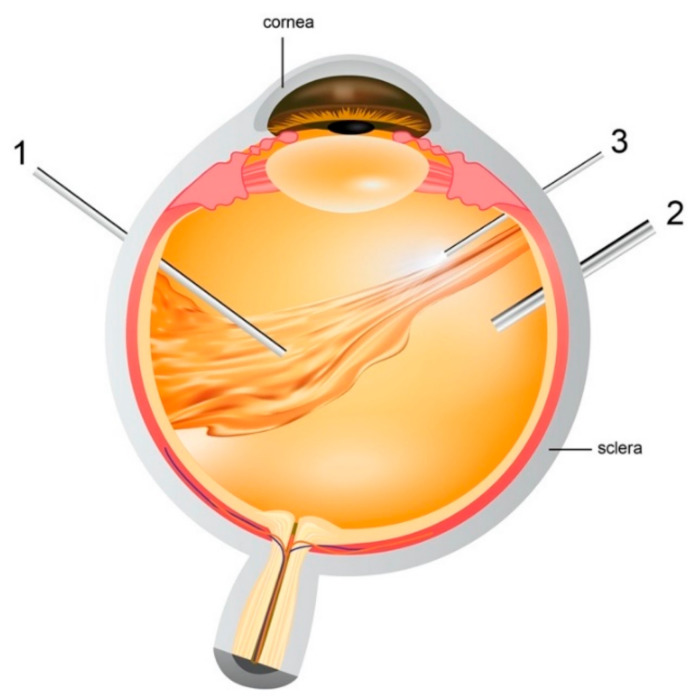
Pars plana vitrectomy procedure- (1) Vitrector: cuts the vitreous gel and allows suction of native vitreous (2) Infusion pipe: allows vitreous replacement with artificial substitute (3) light pipe: to illuminate the eye for the surgeons to explore the vitreous cavity and retina easily.

Liquid based substitutes showcase a wider variety of materials and thus have been more immensely explored. The first liquids to be used as a vitreous substitute were balanced salt solution (BSS) and water [44]. Although, they have quite a bit similarity to the native vitreous in terms of refractive index and transparency but they exert no tamponade effect on the retina due to their short residence time and low viscosity [29]. Therefore, BSS is merely used as a temporary agent to hold on to the retina for a few days when other tamponading materials such as silicone oil is removed and also used for intravitreal washes during the vitrectomy procedure (Figure 3).

#### 4.2.2. Perfluorocarbon Liquid (PFCL)

Perfluorocarbons are synthetic, carbon containing compounds in which fluorine atoms replaces all the hydrogen atoms, forming carbon-fluorine bonds [45]. PFCLs are characterized as colorless, clear and odorless liquids with a low viscosity and density twice as that of water. They are hydrophobic and immiscible with a variety of aqueous substances but are excellent carriers of gases like O_2_ and CO_2_ and for this reason they were originally used as blood substitutes [31,34]. Miyamoto et al., in 1984, used PFCLs, namely perfluoroether for the first time as vitreous substitute in rabbit eyes [46]. Subsequently, Chang et al. in 1987, used PFCLs for retinal detachment in patients with advanced proiferative vitreoretinopathy [47]. Three most commonly used PFCLs currently are perfluorodecalin (PFD), perfluoro-tetradecahydrophenantrene and perfluoro-n-octane (PFO). Currently, PFCLs are used only as temporary tamponades for intraoperative procedures to unfold and stabilize the retina [42]. The long-term toxicity of PFCLs limits their use to intraoperative procedures alone. Previous studies demonstrated that PFCLs cannot be used intravitreally for more than 2-4 days as it causes irreversible cell damage to the inferior retina. PFCLs causes mechanical damage to the cells due to their high specific gravity via compression and reach emulsification by 6th post-operative day. PFCLs require a second surgery for their removal if at all they are being used as a short-term substitute due to their likelihood to emulsify and stimulate inflammatory reactions [48,49].

#### 4.2.3. Semifluorinated Alkanes (SFAs)

SFAs also commonly known as fluorinated alkanes or partially fluorinated alkanes (PFAs). Structurally, SFAs consists of a perfluorocarbon chain to which short alkyl chains are attached to either one or both the ends [50]. Physically and chemically they are inert, colorless, have desired refractive index, heavier than water and immiscible with water. However, they showcase a good solubility in PFCLs, silicone oils and hydrocarbons. Moreover, their low specific gravity (1.35 g/ml) compared to PFCLs results in less retinal damage [51]. SFAs can be used as temporary endotamponade agents for upto 2–3 months [52,53]. Major drawbacks associated with the use of SFA includes cataract, emulsification and soft epiretinal membrane [54,55].

#### 4.2.4. Silicone Oil

Silicone oil (SO) is a synthetic polymer pertaining to polydimetilsiloxanes group which consists of differing chain lengths. Since 1960s, SO have been used in vitreoretinal surgery. In mid 1970s, after pars plana vitrectomy progressed, the use of silicone oil also drastically improved and its long-term use as vitreous substitute gained popularity worldwide. Vitrectomy is recommended in case of severe retinal detachment. Although, it got approved by Food and Drug Administration of the United States to be used intravitreally only in 1994 [45]. It is in a liquid form due to absence of chemical crosslinking between the polymer chains.

Silicone oil is of a great commercial interest for long-term vitreous substitute due to its transparency, high surface tension, low specific gravity, ease of removal and low toxicity. It is able to exert a tamponade effect on the superior retina due to its desired characteristics like low specific gravity, high surface tension and miscibility with water [6,11]. Many researches have proposed that the silicone oil is the desired choice as long-term vitreous substitute in case of tractional retinal detachment (TRD) [56], rhegmatogenous retinal detachment (RRD) [57], giant retinal tears and retinal detachment due to proliferative diabetic retinopathy (PRD) [58]. Besides, it is the preferred choice of tamponade agent in patients who have air travel or visiting high altitude places planned post-surgery. Even though, SO would appear to be an ideal substitute for vitreous, its success rate has been reported to be around 70% [31,59].

However, several complications arise with the long-term use of SO as vitreous substitute which includes its emulsification resulting in cataract formation, glaucoma, corneal toxicity, band keratopathy [60,61]. In order to avoid these complications, surgical removal of silicone oil becomes a necessity, although risk of retinal detachment is associated with the removal process. Other drawbacks include, reduced or no tamponade effect in case of retinal breaks in the inferior part of the eye due to its low density which makes it float on the residual fluid in the eye chamber. Besides, due to its hydrophobicity, it makes poor contact with retina and the remaining aqueous fluid in the cavity which restrains it from filling the total vitreous chamber that is essential for the successful closure of retinal breaks. On the other hand, cells in contact with SO might integrate silicone vesicles, which can hinder the transport of metabolites [32]. Therefore, despite possessing desired characteristics for vitreous replacement, it should be used only during desperate needs, when other alternatives fail, ideally as a temporary substitute.

An ideal long-term tamponade agent for vitreous replacement is long overdue in order to overcome the obstacles of the clinically used vitreous substitutes. Therefore, requirements for being an ideal vitreous substitute is briefed out in Table 3.

## 5. New Generation of Biopolymers as Vitreous Substitutes

The quest for an ideal vitreous substitute has led the researchers to design and develop variant polymers that can mimic the native vitreous. Two strategies were followed: firstly, materials with similar molecular structure (transparency, ability to exert pressure, elasticity), secondly, materials mimicking the physiological and chemical functions of the native vitreous (transportation and diffusion of metabolites or drugs, biocompatibility). The first approach led the research towards the use of modified natural polymers. However, studies revealed that mimicking natural vitreous artificially is next to impossible, although certain potential substitutes have been proposed for short-term use for example hyaluronic acid and collagen. Therefore, the research focus has been shifted towards the use of synthetic polymers, that mimic the mechanical and rheological function of vitreous, but are molecularly different in composition from native vitreous such as poly(1-vinyl-2-pyrrolidinone), polyacrylamide, poly(vinyl alcohol), polymethacrylate, polymethacrylamide etc. The following section provides a briefing about the research carried out in obtaining the prospective vitreous substitute tested in the above mentioned two approaches.

### 5.1. Structural Bio-Mimicry: Natural Polymers

Natural polymers such as hyaluronic acid (HA) and collagen constitutes the two major components of the native vitreous and thus have been the obvious first choice as vitreous substitutes. They were thought to be the best prospective as a vitreous substitute due to their excellent biocompatibility. In early 1970s, hyaluronic acid was used as a vitreous replacement, but unfortunately cannot be used as a prolonged vitreous substitute due to their tendency to degrade rapidly [62]. On the other hand, collagen and its derivatives such as gelatin, polygeline and methylated collagen have been investigated for their use as vitreous substitutes, but they demonstrated a rapid decrease in viscosity and also short retention time in the vitreous chamber [63,64,65]. Research was put into creating semi-synthetic polymers as vitreous substitute with crosslinking of sodium hyaluronate formaldehyde with divinyl sulfone and molecules of gellan. Due to its composition and biocompatibility, it can be used as short-term vitreous replacement, but its effectiveness as a long-term substitute still remains under question because of its excessive water solubility [34]. Attempts have been made to increase the degradation time of HA by oxidizing it with sodium periodate (NaIO_4_) to form aldehyde functional group and subsequently crosslinking with adipic acid dihydrazide to form transparent and colorless oxi-HA/ADH hydrogel. Moreover, it exhibits good transparency and tamponade effect intraocularly [66]. Therefore, such substitutes are recommended for maintained longer in vitreous maybe suitable for clinical long-term use.

### 5.2. Functional Bio-Mimicry: Synthetic Hydrogels

The next step towards researching for an ideal vitreous substitute is mostly synthetic polymers. These synthetic polymers are categorized into polymeric hydrogels and smart hydrogels [67]. Hydrogels are formed by crosslinking of hydrophilic polymer chains that swell in aqueous medium [68]. They are the very first biomaterials to be developed for human use [69]. Smart hydrogels are a relatively new class of hydrogels that respond to external stimuli such as temperature, pH, pressure, light, electric fields and chemicals [70]. Till date, smart hydrogels are still on the experimental level, but has a potential to become the next new class of substitutes, although, there are a few major drawbacks such as triggering of the immune system, leading to the inflammation in the retinal and subretinal spaces [71]. Moreover, sterilization is still a problem as heat may destroy their physical and molecular structure [6]. Therefore, optimization of these materials is required for their use as vitreous substitutes.

Most of the vitreous substitutes used currently (silicone oil, gases such as sulfur hexafluoride) are clinically implanted via syringe for minimum invasion [72]. Therefore, an important goal of hydrogel-based biomaterials is the development of injectable hydrogels. These injectable smart hydrogels can be stored, prepared and injected in a liquid state which can gelate in situ via external stimuli [29]. Thermosensitive hydrogels are an attractive class of injectable hydrogels due to their rapid gelation under physiological conditions of the body free of any external chemical treatment [73]. The delivery of biomaterials to the target can be achieved easily and less invasively with injectable hydrogels as compared to implantation. Moreover, injectable smart hydrogels can easily mold itself by taking up the shape of the defects which are not easily accessible to the conventional delivery methods [74,75].

In 2017, Liang et al., experimented with in situ forming synthetic hydrogels [76]. The study combined polymethacrylamide (PMAM) and polymethacrylate (PMAA) to produce copolymers and introduced thiol groups for reversible crosslinking using bis-methacryloyl cystamine (BMAC). Although, the in-situ gel exhibited good transparency and good biocompatibility at low BMAC concentration, it was observed that with the increase in BMAC concentration, biocompatibility decreases and also acrylamides are carcinogenic and toxic. In another approach, a commercial substance named Healaflow® was tested as potential vitreous substitutes in a rabbit model [77]. It is a synthetic hyaluronic acid hydrogel crosslinked with BDDE (1.4-Butanediol diglycidyl ether), with similar physiological properties as that of native vitreous. It is designed for glaucoma surgery as a space-filler and to restrict postoperative fibrosis. The hydrogel was well tolerated by retina and did not impact the morphology and function of the retina negatively. The authors did not notice any toxicity and histological changes, except the upregulation of glial acidic fibrillary protein (GFAP). It was observed that the retention time was of few weeks, implying its short-term use. Further work suggested by the authors was to extend the structural integrity of the gel for its long-term use.

Smart hydrogels present the future prospect for developing an ideal vitreous replacement. Some of the early examples of smart hydrogels in vitreous substitution include WTG-127 [78] and Pluronic F127 [79]. However, both had their limitations and drawbacks. Therefore, in 2016, Santhanam et al., synthesized a two-component biomimetic hydrogel composed of gellan and PMAM-co-MA), with thiol side groups [80]. It was observed that the hydrogel mimics the physical and optical properties of the native vitreous. Additionally, the hydrogel did not lose its structural integrity before degradation for up to 4 weeks in vitro. More importantly, the two components of the hydrogel provided room for tuning the swelling and mechanical properties, alongside optimizing the transition temperature between 38–41 °C to procure biocompatible hydrogels. Preliminary in vivo studies carried out by the authors revealed no adverse effects on the rabbit’s retinal structure and function and thus indicating its potential suitability as vitreous substitute, though further in vivo experiments need to be carried out. A few months ago, in 2020, Laradji et al., continued the previous work on two component biomimetic hydrogel and reported the four month long preclinical evaluation in rabbit model [81]. The authors evaluated two formulations and observed that both the formulations exude thermosensitive behavior and both exhibit biocompatibility with different cell lines such as ARPE-19 cells, 3T3/NIH cells and primary porcine RPE (ppRPE) cells. Moreover, preclinical evaluation showed no signs of inflammation in the rabbit’s anterior eye segment. Additionally, rabbit’s corneas also seemed transparent. However, at the surgical site of lens, partial opacities were observed using slit lamp but the lens in total was clear. Besides, there was no observed sign of cataract formation in any of the rabbits during the four-month post-operative period. The current preclinical results suggest that this biomimetic hydrogel can be a promising alternative to the commercially available materials, although further assessment of the hydrogel is in progress.

High water absorbent hydrogels have gained popularity and were thought to be a good vitreous substitute. Therefore, recently in 2018, Wang et al., developed a supramolecular binary copolymer PNAGA-PCBAA hydrogel by copolymerizing N-acryloyl glycinamide (NAGA) and carboxybetaine acrylamide (CBAA) [82]. It exudes characteristics similar to native vitreous, such as antifouling, antifibrosis, refractive index, modulus, stability. It also exhibits self-healability, network recoverability and shear-thinning behavior [82]. It appears that this supramolecular copolymer hydrogel not only can be a permanent vitreous substitute, but also can be of use post eye surgery, as an intraocular filling for inner tissue prevention in the eyes.

**Figure 4:**
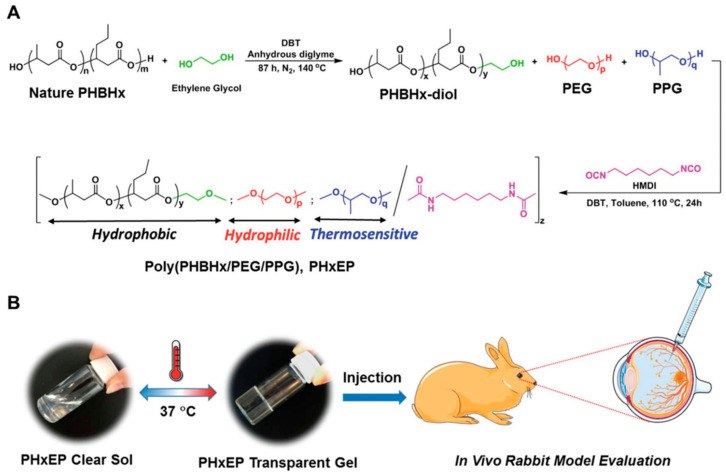
(**A**). Schematic illustration of the synthetic route of poly(PHBHx/PEG/PPG urethane); (**B**). hydrophobic PHBHx content is varied within the hydrogel, and that can change the gel properties from cloudy to transparent at 37 °C. The gels are then tested for in vivo eye application. Image adapted from Loh et al., (2019) and reprinted with permision from Royal society of chemistry.

Recently in 2019, a group of researchers developed poly[(R)-3-hydroxybutyrate-(R)-3-hydroxyhexanoate] (PHBHx)-based polyurethane thermogel named PHxEP (poly(PHBHx/PEG/PPG urethane) [83]. PHBHx shows good machinability, physiochemical properties and biocompatibility. Moreover, it can also degrade into natural, safe and nontoxic components. The PHxEP hydrogel exhibits high light transmittance and is suitable for vitreous substitute. Besides, the hydrogel could maintain transparency in vivo and there was no sign of inflammation in the retinal structure of rabbit’s eye for over 6 months (Figure 4). Therefore, the biocompatible PHBHx based thermogels could form a new generation transparent injectable vitreous replacement.

### 5.3. Future Perspective: Collodial Solution Application in Vitreous

Recent advancements in nanotechnology helps in encapsulating and delivering small molecules to the ocular tissues effectively as compared to the conventional drug delivery systems such as topical administration, subretinal injections, intravitreal injections and oral medicines. All the conventional delivery methods results in poor bioavailability of the drug that reaches the retinal layers [84]. According to the reports for ocular drug delivery, larger sized particles (>1 µm) may potentially cause irritation and foreign body sensation in the ocular region [85]. Therefore, drug filled nanoparticles seem to be a potential solution to reduce the foreign body sensation and irritation in the eye due to their smaller particle size ranging from 1 to 1000 nm. In addition, nanocarriers can treat ocular diseases by enhancing bioavailability of topical administration, achieving tageted delivery, controlled release, lower doseage requiremement, high drug retention and ameliorate therapeutic efficacy [86,87,88].

Recently, polymers were used to develop degradable nanoparticles (NPs) for ocular drug delivery such as liposomes, chitosan nanoparticles, dendrimer, gelatin nanoparticles and poly lactic-co-glycolic acid (PLGA) nanoparticles [89]. The size and charge of these nanoparticles can be tuned to target the desired area in the anterior or posterior region of the eye [86]. These nanoparticles can be modified further to treat vitreous related pathologies in the near future. In 2018, Kabiri et al., developed an in-situ forming, stimuli responsive, nanoparticle-laden hydrogel for sustained release of the drugs into the aqueous humor of the eye as there is a high need for transcorneal drug delivery vehicles [90]. The hydrogel composes of hyaluronic acid and methylcellulose, while the nanoparticles are composed of poly(ethylene oxide) (PEO) and poly(lactic acid) (PLA). The efficacy of nanoparticles loaded with cannabigerolic acid (CBGA) was tested in whole eye experiments and showcased over 300% increase in transcorneal penetration than control formulations. Moreover, the formulation can coat the cornea via blinking of the eyelid and can be used an an eye drop administered immediately prior to the bedtime of the patients.

Intravitreal administration of drugs is limited due to the fast clearance of drugs leading to the need to inject the therapeutic formulations in the vitreous humor every 4–6 weeks to maintain high efficacy. Therefore, to increase the efficacy of the intravitreally administered drugs, it is important to understand their distribution pattern invitreally. Hence, in 2020, Thakur et al., experimented with hyaluronic acid-agar based hydrogel, which is considered to be an ideal artififcial vitreous hydrogel due to its compositional similarity to the native vitreous [91]. However, the drug or nanoparticle migration in this hydrogel is unclear, therefore this study focussed on the viscoelastic behaviour of the gel and nanoparticle and drug migration within the gel compared to the bovne vitreous humor. It was found that low viscosity HA-agar hydrogels are most similar to the freshly excised bovine vitreous in terms of viscoelasticity and nanoparticle and drug migration.

## 6. Conclusions and Future Prospective

The vitreous exhibits distinct biochemical, biomechanical and biophysical properties which helps in maintaining the growth and development of the eye. Various pathological conditions such as age-related pathologies, posterior vitreous detachment, diabetic retinopathy requires removal and replacement of vitreous. Currently used vitreous substitutes holds various disadvantages, majorly related to the lack of biocompatibility. With the advanced understanding of the structure and function of the native vitreous and of various biomaterials, an ideal vitreous substitute can be developed. Various hydrogels have been synthesized and experimented as vitreous substitutes in the recent years of which smart hydrogels hold a potential candidate for a substitute which both biocompatible and long lasting. Future developments based on degradable nanocarriers might hold considerable promise to become an ideal substitute.

## Figures and Tables

**Table 1:** Components in the vitreous.

Component	Molecule	Function	Ref.
Protein	Albumin (40%) Collagen Type II (60-70%) Type IX (25%) Type V/XI (10%) Type IV (<10%)	Provides framework and structure to the vitreous	[6]
GAG	Hyaluronic acid Chondroitin sulfate -Versican -Type IX collagen Heparan sulfate	Determine the viscosity of the vitreous body Provide structural integrity to the tissue Maintains adequate distance between the collagen fibrils	[8,9]
Metabolites	Glucose Lactic acid Ascorbic acid Fatty acids Amino acids	Sustain metabolic activity Helps in hyalocytes proliferation Potential antioxidant Cellular maintenance Cellular maintenance	[11]
Cells	Hyalocytes Fibroblasts Macrophages	Formation of vitreous matrix and its maintenance Regulation and degradation of matrix and cells	[12]

**Table 2:** Overview of the vitreous substitutes used clinically.

Substance	Advantage	Disadvantage	Ref.
Air	Chemically inert, inexpensive, colorless, non-toxic	Short residence time, low refractive index (1.0008)	[33,34]
Gases (SF_6_, C_3_F_8_)	Colorless, odorless, non-toxic	Short residence time; cataract; problems due to increased IOP; awkward face down position after surgery	[38,41,42,43]
Physiological Solutions	Transparent, desired refractive index	Short residence time	[29]
PFCL	Transparent, moderate surface tension	Only intraoperative use is recommended	[48,49]
SFA	Inert, colorless, desired refractive index	Low specific gravity; cataract; emulsification; formation of epiretinal membrane	[53,54,55]
Silicone Oil	Low specific gravity; high surface tension; transparent	Cataract; glaucoma; corneal toxicity; silicone retinopathy; second surgery for its removal; no tamponade in retinal breaks in inferior part of the eye	[6,60,61]

**Table 3:** Characteristics of an ideal vitreous substitute [29,34].

Requirement
Transparent and clear
High water content
Easy to inject and extract through a small syringe
Refractive index and density similar to the natural vitreous
Chemically inert
Biocompatible
Nontoxic
Maintain IOP within the physiological range
Allow easy transport of ion and electrolytes
